# Prevalence of alcohol consumption in emergency presentations: Novel approach using two biomarkers, ethanol and phosphatidylethanol

**DOI:** 10.1111/dar.13534

**Published:** 2022-08-31

**Authors:** Cate M. Cameron, Kim Vuong, Brett McWhinney, Anna Zournazi, Silvia Manzanero, Jacelle Warren, Gary Mitchell, Victoria McCreanor, Kirsten Vallmuur, Tegwen Howell, Jacobus P. J. Ungerer

**Affiliations:** ^1^ Jamieson Trauma Institute Royal Brisbane and Women's Hospital, Metro North Health Brisbane Australia; ^2^ Australian Centre for Health Services Innovation and Centre for Healthcare Transformation Queensland University of Technology Brisbane Australia; ^3^ Chemical Pathology, Pathology Queensland, Queensland Health Royal Brisbane and Women's Hospital, Metro North Health Brisbane Australia; ^4^ School of Clinical Sciences, Queensland University of Technology Brisbane Australia; ^5^ Royal Brisbane and Women's Hospital Metro North Health Brisbane Australia; ^6^ School of Medicine University of Queensland Brisbane Australia; ^7^ Remote Resolve Brisbane Australia; ^8^ Faculty of Biomedical Science University of Queensland Brisbane Australia

**Keywords:** alcohol, emergency service, epidemiology, hospital, prevalence

## Abstract

**Introduction:**

The aim was to determine the prevalence of alcohol‐related presentations to an emergency department (ED) in a major Australian hospital, through a novel surveillance approach using two biomarkers, blood ethanol and phosphatidylethanol (PEth).

**Methods:**

Observational study using secondary testing of blood samples collected during routine clinical care of ED patients presenting to the Royal Brisbane and Women's Hospital in Queensland, Australia, between 22 January and 2 February 2021. Data were collected from 1160 patients during the 10‐day study period. The main outcomes were the prevalence of acute alcohol intake, as determined by blood ethanol, and recent use over 2–4 weeks, as determined by PEth concentrations, for all ED presentations and different diagnostic groups.

**Results:**

The overall prevalence for blood ethanol was 9.3% (95% confidence interval [CI] 7.8%, 11.1%), 5.3% for general medical presentations, increasing four‐fold to 22.2% for injury presentations. The overall prevalence of PEth positive samples was 32.5% (95% CI 29.9%, 35.3%) and 41.4% for injury presentations. There were 263 (25.3%) cases that tested negative for acute blood ethanol but positive for PEth concentrations indicative of significant to heavy medium‐term alcohol consumption.

**Discussion and Conclusions:**

This novel surveillance approach demonstrates that using blood ethanol tests in isolation significantly underestimates the prevalence of medium‐term alcohol consumption in ED presentations. Prevalence of alcohol use was higher for key diagnostic groups such as injury presentations. Performing periodic measurement of both acute and medium‐term alcohol consumption accurately and objectively in ED presentations, would be valuable for informing targeted public health prevention and control strategies.

**Key Points**

- The impact of alcohol intake on presentations to the emergency department reveals a significant public health problem. In addition to the short‐term harmful effects of intoxication, regular heavy alcohol consumption has been linked with cancers, other diseases and injuries.
- The biomarker phosphatidylethanol, a reliable measure of medium‐term alcohol intake, is useful for public health research using a novel surveillance approach via secondary testing of blood samples.
- This study opens opportunities for surveillance, monitoring and informing health‐care provision across many health settings, by eliminating self‐reporting and including measurements beyond immediate blood ethanol levels.

## 1 INTRODUCTION

The importance of identifying unhealthy alcohol use was highlighted in a recent supplementary paper on the Guidelines for the Treatment of Alcohol Problems, commissioned by the Commonwealth Government of Australia [1]. Between 2015 and 2020, the most common drug‐related hospitalisation in Australia was alcohol‐related [2]. The burden of alcohol‐related presentations to emergency departments (ED) is also widely reported in the literature [3, 4, 5]. This includes increased demand on hospital resources, burden and risk to clinical staff as well as harm or injury to the individual. Furthermore, regular harmful alcohol consumption has been linked to a number of diseases including cancers, diabetes, cardiovascular and digestive diseases, as well as increasing risk of injury and infectious diseases from risk taking behaviours while intoxicated [6].

There is a need to monitor and understand the prevalence of acute and medium‐term alcohol consumption using objective measurements such as direct markers of alcohol. The use of screening tools and self‐report measures has known limitations such as the limited sensitivity due to intentional underreporting of drinking behaviours or inaccurate recollection of events [7, 8, 9]. The use of direct markers of alcohol would address the abovementioned limitations of screening tools and self‐report measures and would enable any changes in consumption, for example, related to the COVID‐19 pandemic, to be identified early and addressed. However, in Queensland, Australia, there is no routine collection or objective measurement of alcohol consumption in patients presenting to EDs, and is legally prohibited unless required by police for traffic‐related incidents [5, 10].

A biomarker commonly used to detect alcohol consumption is blood ethanol, however, due to its relatively short half‐life, it is only appropriate for detecting alcohol intake in recent hours [11]. A relatively new biomarker phosphatidylethanol (PEth) can detect alcohol concentrations in blood around 2–4 weeks after consumption [12, 13]. PEth is an abnormal cellular membrane phospholipid which is only formed in the presence of ethanol [12], accumulating after repeated drinking, with more PEth formed in the blood as more alcohol is consumed [13]. This makes PEth suitable for the differentiation of excessive alcohol consumption and moderate drinking. Since PEth is only formed in the presence of ethanol, it has high sensitivity and is a reliable and specific method for determining alcohol‐use [15]. A recent review on biomarkers available for the detection of acute and chronic ethanol use found that indirect (carbohydrate‐deficient transferrin, mean corpuscular volume) biomarkers, when compared to direct biomarkers, such as PEth, lacked sensitivity and specificity for the detection of unhealthy alcohol consumption [16].

Previous studies have used PEth to determine alcohol consumption in a variety of settings/populations, such as in patients with liver disease [17], liver transplant recipients [18] or those in an outpatient treatment setting [19]. However, no studies have used PEth and blood ethanol to determine immediate and ongoing alcohol consumption in patients presenting to an ED.

This research aimed to: (i) describe the prevalence of alcohol consumption; and (ii) test the ethical and practical processes required to conduct a prevalence study of alcohol consumption, adopting a novel approach of using blood samples collected during routine care in ED presentations with two biomarkers, blood ethanol and PEth. Monitoring and understanding acute and medium‐term (or more ‘regular/average’) alcohol‐use will inform targeted public health prevention and control strategies.

## 2 METHODS

### 2.1 Study design

This observational study, assessing the Prevalence of Alcohol Consumption in Emergency presentations (PACE), was conducted at the Royal Brisbane and Women's Hospital (RBWH) in Queensland, Australia, using secondary testing of routinely collected blood samples from the Emergency and Trauma Centre [20], which will hereafter be referred to as ED. The RBWH is Queensland's largest hospital with over 82,000 ED presentations annually [21]. Approximately 40% of patients who present to RBWH ED have blood samples collected as part of routine clinical care [20].

During the study period, blood samples routinely sent to Pathology Queensland were intercepted and an aliquot separated and frozen without patient or treating clinician's knowledge [20]. To avoid any impact on patient care, the samples were batch tested after 3–4 months. De‐identified test results, and clinical data extracted from the ED Information System, were received by the study team for analysis.

### 2.2 Biomarker testing

Ethanol was tested using serum samples. Blood ethanol concentrations were measured with an enzymatic assay on a Beckman Coulter analyser [22]. To quantify PEth, a validated ultra‐performance liquid chromatography tandem mass spectrometry (UPLC‐MS/MS) method was used in the range of 0–2000 μg/L [15, 20]. PEth is extracted from whole blood by protein precipitation with 2‐propanol. The coefficient of variation is ≤8%; recoveries are 98–102%, the limit of the blank is 1 ug/L, limit of detection 1.3 ug/L and limit of quantification (20% CV) of 2.1 ug/L.

The fatty acid side chains of PEth varies, resulting in several different PEth molecule species or homologues [12]. The homologue 16:0/18:1 was found to be the dominant species and showed excellent correlation with total PEth (*r* ^2^ = 0.98, *p* < 0.001) and was therefore selected as the biomarker for measuring PEth in this study. Further details on PEth testing are provided in the study protocol [20].

### 2.3 Ethics

Ethics approval was obtained by the RBWH Human Research Ethics Committee (LNR/2019/QRBW/56859). A waiver of consent was obtained in accordance with section 2.3.10 of the National Statement on Ethical Conduct in Human Research. Additionally, in Queensland, under Section 280 of the *Public Health Act*, Director General approval is required for the release of confidential information for the purposes of research. *Public Health Act* approval was obtained for the release of the patient demographic and clinical data (PHA 56859).

### 2.4 Data collection

Blood samples were identified over 10 days between 22 January and 1 February 2021 across the full 24 h, which included the 4‐day Australia Day festive long weekend. At the time the local area was not in a COVID‐19 lockdown, public restrictions were minimal and case levels very low. COVID restrictions were further lifted on 22 January 2021. Masks were no longer mandated, ticketed venues were 100% seated capacity, and weddings and funerals allowed numbers up to 200 people. State borders opened in December 2020 with quarantine restrictions for people arriving from interstate hotspots and overseas. The total known active cases in Queensland on 22 January 2021 was 23 dropping to 6 active cases on 1 February 2021 [23].

Demographic and clinical data from all patients who presented to the ED during the study period were extracted from the ED Information System. Demographic information included gender, age, identification as Aboriginal and/or Torres Strait Islander and postcode of usual residence. Clinical information included the principal diagnosis code (International Classification of Diseases, Tenth Edition, Australian Modification; ICD‐10‐AM), the date and time of presentation, mode of arrival, triage priority, the free text presenting problem, place of discharge or admission, and whether the patients' health care was compensable under a transport, workers or liability insurance scheme. Blood ethanol and PEth test results were provided by Pathology Queensland with the Study ID for linkage.

### 2.5 Data analysis

**FIGURE 1:**
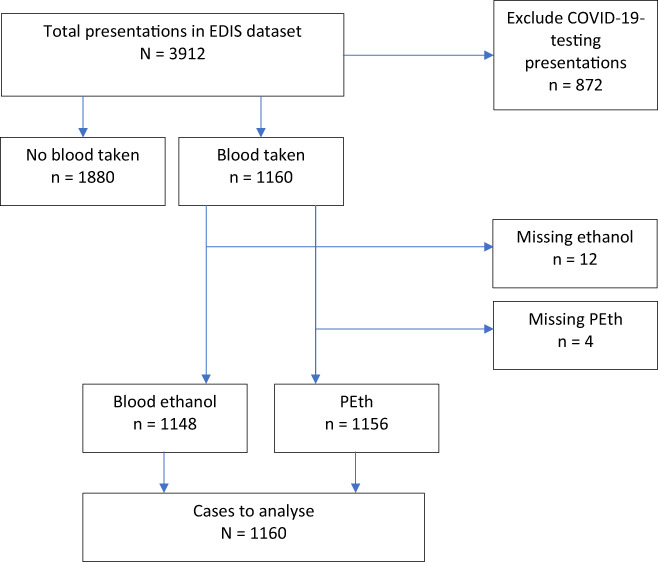
Flow diagram of case inclusion for the prevalence of alcohol consumption in emergency presentations study sample. EDIS, Emergency Department Information System; PEth, phosphatidylethanol

Data management and analysis was conducted using SPSS 27.0.1.0 and SAS 9.4. Missing data and unexpected outliers were rechecked for completeness with original data sources via the relevant data custodians. Descriptive statistics included frequencies, percentages, means, SDs and medians. COVID‐19 testing presentations were excluded from analysis (Figure 1).

Sample representativeness was determined by comparing the demographic and clinical characteristics of patients with and without blood collections during the study period, using chi‐square and *t* tests as appropriate. To consider the generalisability of the findings, demographic and clinical characteristics of all the patients who presented to the RBWH ED during the study period were compared to publicly available Queensland and National ED data for the same financial year, using chi‐square tests [24].

To examine the impact of the public holiday weekend on prevalence estimates, the sampling period was divided into two periods, the first 5 days included the public holiday weekend and the remaining days being a non‐festive period. Both periods included weekend days.

Study sample presentations were categorised into four groups for analysis based on the primary ICD‐10‐AM diagnosis: (i) general medical (broadly including diagnoses such as different types of chest pain, abdominal pain, genitourinary and digestive diagnoses); (ii) alcohol‐related; (iii) injury‐related; and (iv) mental health‐related (Table 1).

**TABLE 1:** Demographic and clinical characteristics of the prevalence of alcohol consumption in emergency presentations study sample (*n* = 1160)

	*n* (%)
**Demographics and clinical information**	
Gender	
Female	630 (54.3)
Male	530 (45.7)
Age, mean (SD)	48 (*21*)
Age groups, years	
<18	23 (2.0)
18–44	524 (45.3)
45–64	314 (27.1)
65+	297 (25.6)
Missing = 2	
Aboriginal and/or Torres Strait Islander	
No	1106 (95.3)
Yes	49 (4.2)
Missing = 5	
Triage code^a^	
1–3	820 (70.7)
4 and 5	340 (29.3)
Mode of arrival	
Ambulance (road and air)	617 (53.2)
Walk‐in	513 (44.2)
Emergency examination authorities (ambulance/police)	30 (2.6)
**Primary diagnosis categories**	
General medical^b^	**921 (79.4)**
Symptoms, signs not elsewhere classified	*275 (23.7)*
Abdominal pain (*n* = 69)	
Non‐cardiac chest pain (*n* = 37)	
Circulatory system	*118 (10.2)*
Possible cardiac chest pain (*n* = 42)	
Genitourinary system	*105 (9.1)*
Urinary‐related (*n* = 58)	
Digestive system	*105 (9.1)*
Constipation/gastrointestinal haemorrhage/appendicitis (n = 37)	
Other general medical	*318 (27.4)*
Eye/ear and mastoid/skin and subcutaneous (n = 91)	
Respiratory (*n* = 45)	
Infectious and parasitic (*n* = 33)	
Endocrine, metabolic (*n* = 26)	
Musculoskeletal (*n* = 26)	
Injury/alcohol‐related	**202 (17.4)**
Injury^c^	*186 (16.0)*
Alcohol‐related^d^	*16 (1.4)*
Mental health^e^	**37 (3.2)**

Abbreviation: ICD‐10‐AM, International Classification of Diseases, Tenth Edition, Australian Modification.

^a^ Triage Codes 1‐5. 1 = life‐threatening illness or injury requiring immediate attention, 5 = non‐emergency health concern.

^b^ Subcategories for general medical were reported for the most frequent presentation groups.

^c^ All ICD‐10‐AM codes in Chapter 19 were included with the exception of codes T80‐T88 related to complications of surgical and medical care. Codes W46, W54, W57 and X84 were also included in the injury categories (external causes of injury, i.e., needle, animals, insects).

^d^ ICD‐10‐AM codes included were F10.0, F10.3, K29.2, R78.0, Y90 and Z72.1.

^e^ ICD‐10‐AM codes included were in the range F01‐F99 excluding F10.0 and F10.3.

Blood ethanol and PEth prevalence were presented using predefined categories described below [20], with 95% confidence limits based on the Wilson Method [25], for the total sample and each diagnostic group. The strength of the association between ethanol and PEth measurements, in their continuous form, was examined using non‐parametric Spearman's Rho correlation coefficient.

Ethanol results were converted to blood alcohol concentration (BAC) equivalents, gram % (g%) for reporting. The BAC classifications were guided by recommended cut‐offs based on the effects a ‘healthy person’ is likely to experience [26] and included: light consumption (BAC 0.007 to <0.05 g%); moderate consumption (0.05 to <0.15 g%); significant consumption (BAC 0.15 to <0.30 g%); and potential coma or death (BAC 0.3+ g%). The ‘No consumption’ cut‐off had a BAC of <0.007 g% (0 to <1.6 mmol/L) due to some uncertainty in measurements of ethanol below 1.6 mmol/L.

PEth levels were categorised into three groups: light or no consumption (<20 ng/mL); significant consumption (20–200 ng/mL) and heavy consumption (>200 ng/mL) [14]. Significant consumption (PEth levels between 20 and 200 ng/mL) indicates an average moderate level of drinking or possible isolated binge drinking in recent weeks. Whereas heavy consumption (PEth levels of >200 ng/mL) indicates likely frequent heavy drinking with these levels unlikely due to isolated binge drinking [14].

## 3 RESULTS

A total of 3912 patients presented to the ED during the study period. COVID‐19 testing presentations (*n* = 872) were removed. From the remaining 3040, 1160 (38.2%) patients had blood samples intercepted for this study. Absent or insufficient samples resulted in 12 missing ethanol tests and 4 missing PEth tests (Figure 1).

### 3.1 Sample characteristics, representativeness and generalisability

The mean age of the PACE sample (*n* = 1160) was 48 years (SD = 21), with more females than males, and 4.2% identifying as Aboriginal and/or Torres Strait Islander (Table 1). The most common mode of arrival was by ambulance (road or air, 53.2%) followed by walk‐ins (44.2%). Most presentations had a triage code between 1 and 3 (70.7%). Triage codes range from 1 to 5 with a triage code 1 being most severe, representing a life‐threatening illness or injury requiring immediate attention, to a triage code 5 being the least severe, representing a non‐emergency health concern to be treated within 2 h. Most presentations had a primary diagnosis in the general medical category (79.4%), 16.0% had an injury‐related primary diagnosis, 1.4% had an alcohol‐related primary diagnosis and 3.2% were diagnosed as mental health‐related presentations.

Chi‐square analyses showed differences in the characteristics of the study sample (*n* = 1160), compared with the remaining ED patients not included during the study period (*n* = 1880). The study sample included more females than the remaining ED presentations (study sample: 54.3% female vs. remaining ED: 48.3% female); more patients aged 45 years and above (study sample: 52.7% 45+ years vs. remaining ED: 37.3% 45+ years); and more patients with higher priority triage scores, with 70.7% of the study sample being triage category 1–3, compared to 50.1% of remaining ED patients being triage category 1–3 (all *p* < 0.01) (results not shown).

There were no statistically significant differences in the proportions for age, gender, Indigenous status or triage category, when comparing the total RBWH ED presentations during the study period (*N* = 3040) with corresponding Queensland and National ED data for the same financial year (all *p* > 0.05) (results not shown) [24].

### 3.2 Ethanol prevalence

The overall prevalence of blood ethanol positive samples was 9.3% (95% CI 7.8%, 11.1%). Of these, 6.9% were found to have moderate or significant levels of ethanol (BAC 0.05 to <0.30 g%) and 1.0% had a level that could result in potential coma or death (Table 2). While there was no statistically significant difference in the proportion of ethanol positive samples collected over the long weekend festive period compared to samples collected on the non‐festive days (6.6% non‐festive vs. 9.2% festive; *p* = 0.096), the samples collected in the festive period had significantly more ethanol concentrations above BAC 0.1 g% (2.72% non‐festive vs. 6.18% festive; *p* = 0.004, results not shown).

**TABLE 2:** Prevalence of ethanol and phosphatidylethanol concentrations in prevalence of alcohol consumption in emergency presentations study sample (*N* = 1160)

	*N* (%) [95% CI]
Ethanol concentration	
No consumption (BAC <0.007 g%)	1041 (90.7) [88.9%, 92.2%]
Light (BAC 0.007 to <0.05 g%)	17 (1.5) [0.9%, 2.4%]
Moderate (BAC 0.05 to <0.15 g%)	40 (3.5) [2.6%, 4.7%]
Significant (BAC 0.15 to <0.30 g%)	39 (3.4) [2.5%, 4.6%]
Potential coma/death (BAC 0.30+ g%)	11 (1.0) [0.5%, 1.7%]
Missing	12
PEth concentration	
No consumption/light (0 to <20 ng/mL)	780 (67.5) [64.7%, 70.1%]
Significant (20 to <200 ng/mL)	236 (20.4) [18.2%, 22.8%]
Heavy (200+ ng/mL)	140 (12.1) [10.4%, 14.1%]
Missing	4

*Note*: Proportions calculated on non‐missing data.

Abbreviations: BAC, blood alcohol concentration; CI, confidence interval; PEth, phosphatidylethanol.

Examination of the primary diagnosis categories showed the lowest prevalence of blood ethanol in general medical patients with only 5.3% (95% CI 4.0%, 6.9%) positive samples (Table 3). Whereas patients presenting following injury had over four times the prevalence, with 22.2% (95% confidence interval [CI] 16.8%, 28.7%) of cases positive for ethanol. Of these, 10.8% had significant levels of ethanol (BAC 0.15 to <0.30 g%) (95% CI 7.7%, 16.1%) and 1.6% were at a level that could result in potential coma or death (95% CI 0.6%, 4.7%) (results not shown). Alcohol‐related presentations, based on ICD‐10‐AM coded primary diagnosis were few (*n* = 16) and though caution is required due to the small numbers, ethanol prevalence was, as expected, high for this group (81.2%; 95% CI 57.0%, 93.4%).

**TABLE 3:** Prevalence of ethanol and phosphatidylethanol positive samples in prevalence of alcohol consumption in emergency presentations study for the total sample and by diagnostic categories

	Total sample, *N* = 1160	General medical (*n* = 921)	Alcohol (*n* = 16)	Injury (*n* = 186)	Mental health (*n* = 37)
	*N* (%) [95% CI]	*N* (%) [95% CI]	*N* (%) [95% CI]	*N* (%) [95% CI]	*N* (%) [95% CI]
Ethanol concentration					
Negative (BAC <0.007 g%)	1041 (90.7) [88.9%, 92.2%]	863 (94.7) [93.1%, 96.0%]	3 (18.8) [6.6%, 43.0%]	144 (77.8) [71.3%, 83.2%]	31 (86.1) [71.3%, 93.9%]
Positive (BAC 0.007+ g%)	107 (9.3) [7.8%,11.1%]	48 (5.3) [4.0%,6.9%]	13 (81.2) [57.0%,93.4%]	41 (22.2) [16.8%, 28.7%]	5 (13.9) [6.1%, 28.7%]
Missing	12	10	0	1	1
PEth concentration					
Negative (0 to <20 ng/mL)	780 (67.5) [64.7%, 70.1%]	646 (70.4) [67.4%, 73.3%]	0 (0.0)	109 (58.6) [51.4%, 65.4%]	25 (67.6) [51.5%, 80.4%]
Positive (20+ ng/mL)	376 (32.5) [29.9%, 35.3%]	271 (29.6) [36.7%, 32.6%]	16 (100.0) [80.6%,100.0%]	77 (41.4) [34.6%, 48.6%]	12 (32.4) [19.6%, 48.5%]
Missing	4	4	0	0	0

*Note*: Proportions calculated on non‐missing data.

Abbreviations: BAC, blood alcohol concentration; CI, confidence interval; PEth, phosphatidylethanol.

### 3.3 PEth prevalence

Overall, nearly one‐third of the samples revealed PEth levels indicative of significant to heavy alcohol consumption in the past 2–4 weeks (Table 2). Just over 12% (95% CI 10.4%, 14.1%) of the total sample revealed heavy alcohol consumption with at least 200 ng/mL. Unlike the acute ethanol results, there was no difference in the proportion of PEth positive samples or PEth concentrations when comparing samples from patients who presented over the festive long weekend or non‐festive days (*p* = 0.95; results not shown).

Prevalence levels for PEth differed by primary diagnosis group. Like the blood ethanol prevalence, the general medical group showed the lowest proportion of PEth positive presentations (29.6%; 95% CI 36.7%, 32.6%), compared with 41.4% (95% CI 34.6%, 48.6%) in the injury group, and 100% (95% CI 80.6%, 100.0%) for the alcohol‐related presentations (Table 3). In the injury group, almost 20% of patients had heavy alcohol consumption in recent weeks, testing positive for PEth at the highest level of at least 200 ng/mL (19.4%, 95% CI 14.3%, 25.6%). In the alcohol‐related presentations group, 87.5% of cases had a PEth result in the highest category (95% CI 64.0%, 96.5%) (results not shown).

### 3.4 Correlation of ethanol and PEth

To compare the strength of the association between ethanol and PEth results, only complete cases with both test results were examined (*n* = 1144). In their raw continuous form, the two tests showed only moderate correlation (*r*_s_ = 0.29, *p* < 0.05). When the ethanol and PEth measurements were dichotomised as negative or positive and compared (Table 4), the PEth test showed high sensitivity detecting 100% of the samples that were ethanol positive. Additionally, 263 patients who had negative ethanol results tested positive for PEth (25.3%). A quarter of the 263 cases (*n* = 66) showed PEth concentrations greater than 200 ng/mL, indicative of heavy alcohol intake, with a maximum reading of 2372 ng/mL, nearly 12 times higher than the lower limit for heavy consumption (results not shown).

**TABLE 4:** Comparisons between ethanol and phosphatidylethanol positive and negative results for complete pairs of tests, *N* = 1144

	Medium‐term PEth (20+ ng/mL)	
Acute ethanol (BAC 0.007+ g%)	Negative, *n* (%)	Positive, *n* (%)
Negative	775 (74.7)	263 (25.3)
Positive	0 (0.0)	106 (100.0)

Abbreviations: BAC, blood alcohol concentration; PEth, phosphatidylethanol.

## 4 DISCUSSION

Monitoring and understanding alcohol consumption in ED patients is critical to determining the need for public health action and informing targeted prevention and management strategies [27]. This is the first study to use a novel surveillance approach using routinely collected biospecimens and two biomarkers to objectively measure alcohol exposure in patients presenting to the ED. Our prevalence estimates, based on blood ethanol and PEth concentrations, demonstrate that using ethanol tests in isolation can substantially underestimate alcohol consumption in patients attending the ED.

The finding of only moderate correlation between the ethanol and PEth test results was not surprising given ethanol measures blood alcohol concentrations within hours of consumption and PEth measures alcohol consumption over 2–4 weeks. Understanding levels of ongoing alcohol use is important from a medical and public health perspective as it is a known causal factor in more than 200 different disease and injury conditions contributing to considerable health burdens [28].

While just under 10% of patients had positive blood ethanol concentrations, nearly one‐third of all patients had PEth levels indicative of significant to heavy alcohol consumption in the previous month. This was higher again for some patient groups such as those presenting with an injury. Additionally, 25% of patients who were positive for significant to heavy PEth concentrations would have been missed from estimates of prevalence of alcohol use if the traditional ethanol tests were used in isolation. Of greater concern, is that a quarter of those potentially missed cases (*n* = 66) showed PEth concentrations that were greater than 200 ng/mL with a maximum reading of 2372 ng/mL, nearly 12 times higher than the lower limit for heavy consumption.

The current study findings would suggest prevalence testing using BACs are subject to variation depending on the chosen sampling period, with festive days showing increased acute ethanol concentrations. Whereas the PEth testing showed no differences over the festive and non‐festive sampling periods. With UPLC‐MS/MS methods, whole blood PEth is sensitive (>98%) and specific (100%) for identifying recent moderate or heavy alcohol use. PEth testing may provide more reliable prevalence estimates for periodic surveillance measures and public health planning.

### 4.1 Strengths, limitations and ethical considerations

The PACE study demonstrates the benefits of secondary testing of routinely collected biospecimens to objectively measure prevalence of alcohol consumption in patients presenting to the ED and to provide important public health surveillance data. Considerable time was spent with the research team and ethics committee working together to address potential ethical challenges for this study.

The secondary testing of existing blood specimens with a waiver of consent, was considered the most ethical way to objectively measure prevalence while maintaining study validity and minimising harm to patients. A waiver of consent was critical to avoid known biases, enabling testing of all possible samples. Low consent rates typically plague studies of sensitive topics, such as alcohol and other drug use, leading to consent bias [29, 30]. The secondary use of existing biospecimens is an improvement on previously reported data collection methods, such as using the Alcohol Use Disorders Identification Test, that often require dedicated staff on‐site in the ED for 24‐h time periods to conduct patient screening, consenting and interviews [30, 31]. Self‐reported responses are subjective and can be affected by social desirability biases where participants respond with what they think are acceptable and desired by the interviewers [32, 33].

ED staff were blinded to the data collection period to prevent the occurrence of a Hawthorne effect [34, 35], where staff may unconsciously or consciously change usual care and increase the frequency of blood testing if they were aware the study was being conducted. This was ethically important to minimise potential harm to patients. Additionally, to strengthen ethical separation, samples were identified and stored for batch testing 3–4 months later to ensure results could not affect the clinical management of patients. No test results were provided to clinicians, and researchers only had access to de‐identified data for analysis months after patient presentations. However, because ED staff were not aware of the study and had not requested the blood ethanol or PEth tests through usual documented processes, considerable practical challenges to identify and intercept samples arose for the Pathology staff. To repeat this study or to introduce routine periodic surveillance, these process challenges would need to be addressed.

Unlike other indirect alcohol markers, PEth is only formed in the presence of ethanol, accumulates with increased frequency of consumption, and can distinguish between those abstaining from alcohol, and those with regular moderate and high ethanol intake [13, 36]. Additionally, PEth is considered to be more reliable than other indirect alcohol markers like carbohydrate‐deficient transferrin, gamma‐glutamyl transpeptidase or alanine aminotransferase, as it is not affected by factors such as medications or health problems unrelated to alcohol consumption, such as celiac disease, cancer, thyroid disease and metabolic syndromes [13, 19, 37]. Nevertheless, the interpretation of results should be considered within the limitations of each biomarker used. PEth results will indicate recent ethanol use. It is not a marker for acute ethanol intoxication, for which serum ethanol is a biomarker.

Due to costs of PEth testing and the specialist equipment required, real‐time testing for clinical purposes in the ED is not currently feasible, and in Queensland legislatively prohibited without consent. For monitoring purposes, periodic testing of existing biospecimens may be more feasible than ongoing routine screening. Options for future studies may also include newer techniques using dried blood spot samples. Dried blood spots have recently been shown to: (i) increase sample stability by the drying process preventing the degradation of PEth; (ii) be minimally invasive for collection; (iii) reduce the need for consumables such as sampling tubes, pipette tips and glass vials; and (iv) simplify storage, thus reducing costs [38].

### 4.2 Sample representativeness and generalisability

The results of this study may not be truly representative of all RBWH ED presentations, as the study sample consisted of more females, older patients and higher triage priorities than the remaining patients attending the RWBH ED during the study period. Sampling from several sites across the state is needed to minimise the current potential single site sampling bias and improve generalisability. A larger sample size would be required for more detailed stratification by site and diagnosis groups.

The current study is from a metropolitan ED and therefore findings may not be generalisable to non‐metropolitan areas. During the same financial year, National Alcohol Statistics show that in outer regional and remote regions of Australia, greater proportions of the population consume alcohol above the recommended Australian Adult Alcohol Guidelines (30.3%), compared with major cities (24.5%) [39]. However, there were no statistically significant differences in the demographic characteristics and triage categories of the total RBWH ED presentations during the study period, compared with state and national ED data for the same financial year 2020–2021 [24], suggesting that patients presenting at RBWH ED would be similar to those attending other EDs, not only across Queensland but nationally.

### 4.3 Future research and conclusion

The novel surveillance approach of the PACE study demonstrates the value of using routinely collected biospecimens and both ethanol and PEth biomarkers to determine and better understand alcohol consumption. This study aligns with the recently published Guidelines for the Treatment of Alcohol Problems [1] where the importance of identifying unhealthy alcohol‐use was highlighted. Wider routine application of reliable tests for alcohol consumption is needed for accurate and early detection of alcohol use trends. Periodic sampling to identify patterns geographically and to monitor trends over time is important for detection of harmful alcohol use and to inform clinical practise guidelines and target public health interventions. Finally, a key area for future work is the need for ascertaining population norms for PEth levels which is vital for proper control comparisons.

## AUTHOR CONTRIBUTIONS

Each author certifies that their contribution to this work meets the standards of the International Committee of Medical Journal Editors.

## FUNDING INFORMATION

This study was financially supported by the Jamieson Trauma Institute, Metro North Health.

## CONFLICT OF INTEREST

None to declare.
